# An approach to preschool task design: how narrative reveals the functionality of early math concepts

**DOI:** 10.3389/fpsyg.2026.1911764

**Published:** 2026-07-29

**Authors:** Anastasia Sidneva, Anastasia Lobanova, Elena Vysotskaya

**Affiliations:** Federal Scientific Center for Psychological and Interdisciplinary Research, Moscow, Russia

**Keywords:** counting, early mathematical concepts, executive functions, narrative, preschoolers, task design

## Abstract

Many preschoolers can count, yet formal mastery does not mean that counting is used meaningfully or embedded in other activities. Story-based tasks can reveal whether a child is acting formally, yet the literature lacks clear guidance on how such tasks should be designed. Drawing on CHAT, we identified five principles for constructing narrative-based tasks and developed a task battery designed to assess three levels of counting mastery: formal (targeting only operational mastery of counting), reasonable (addressing orientation to essential conditions of counting), and functional (capturing whether the child can use counting as a tool embedded in another activity). The battery was piloted on 56 older preschoolers (M = 68.9 months, SD = 3.86). To validate the battery, we assessed executive functions (working memory, inhibition, cognitive flexibility) using NEPSY-II and DCCS—expecting formal and reasonable levels to replicate known correlations with early mathematics, while the functional level might show a different pattern. The task battery successfully differentiated among the three levels of counting mastery: performance decreased systematically from formal to reasonable to functional (*p* < 0.001). Performance on tasks assessing formal and reasonable levels correlated with executive functions (Rs = 0.28–0.41, *p* < 0.05), whereas performance on tasks assessing the functional level showed no such correlations, suggesting that functional counting may rely on different mechanisms. The findings suggest that specially constructed story-based tasks may be necessary for assessing whether counting is used reasonably and embedded in another activity. The battery shows preliminary promise for distinguishing between formal and functional mathematical understanding in preschoolers, though further validation is needed.

## 1 Introduction

Parents and educators often take pride in preschoolers who can count to 100 and back, recognize all numerals, and even write them down. But does this really mean the child understands what numbers are for? Often, the same child may struggle to count the chairs in a room or determine whether there are enough plates for all the guests—yet mathematical operations are merely procedures, not tools for solving real-world situations (Gelman and Gallistel, 1978; Fuson, 1992; Sowder, 1989). The problem of distinguishing between formal and functional mastery is well recognized in educational assessment, particularly at the school level, where it is discussed in terms of functional literacy (UNESCO, 1978; OECD, 2023), lower- vs. higher-order thinking (Bloom et al., 1956; Anderson and Krathwohl, 2001), misconceptions (Eleftheriadi et al., 2023), and procedural vs. conceptual knowledge (National Research Council, 2001). In preschool education, however, this distinction has received far less attention, even though manifestations of applying a procedure without understanding its meaning are increasingly observed in young children, which appears to be related to the growing trend toward earlier formal mathematics instruction (Wright et al., 2006; Muthukrishnan et al., 2019).

Within the CHAT tradition, researchers conceptualize formal mastery not merely as a lower level of understanding, but as a specific relationship the child adopts toward the cultural tools to be mastered (Vygotsky, 1978). Children with a formal attitude treat these tools as external prescriptions to be learned and reproduced, without understanding their relevance to reality (Bozhovich, 1980; Sidneva, 2025; Vysotskaya et al., 2025). Formative experiments have shown that this formal attitude reflects the absence of reasonableness—the child does not orient to the essential conditions of the task (Galperin, 1966). The ultimate goal of mastery, then, is not merely reasonable application but the integration of the tool into another activity, where it ceases to be an operation performed upon request and becomes a functional instrument serving broader goals (Galperin, 1966; Nezhnov et al., 2011). Researchers documented specific patterns of formal counting mastery in preschoolers and developed tasks specifically designed to provoke formal solutions, revealing when children apply procedures mechanically rather than meaningfully (Galperin and Georgiev, 1960; Davydov, 1962; Obukhova, 1972).

To detect such formal solutions, tasks need to go beyond requiring a procedure: they must present a meaningful situation in which the child has to decide whether and how to apply counting. Story-based tasks, which embed mathematical operations in meaningful contexts, are well suited for this purpose and are widely used in preschool mathematics education to increase children's interest and engagement (Pollitt et al., 2015; Ginsburg, 2006; Ginsburg et al., 2008; Clements and Sarama, 2014; Chatzaki et al., 2024). In contrast to bare numbers, story problems always include a narrative—that is, a situation described in the task (Koedinger and Nathan, 2004; Carpenter and Moser, 1984; Beloshistaya, 2017). Yet in practice, such tasks are often only formally “decorated” with an everyday or fairy-tale context—the first part of the task, which introduces characters and a setting, is frequently the sole concession to a narrative (Gerofsky, 1996). For example, in counting, addition, and subtraction tasks a scenario is described in which one character gives or receives apples or candies from another, and the child is asked to calculate the result (Verschaffel et al., 2000; Carpenter et al., 1999; Beloshistaya, 2017; Mikhailova and Ioffe, 2003). Such a “formal” narrative does not allow children to fully engage with the task; consequently, they develop strategies to ignore it: they search for keywords (e.g., “in total,” “left over”) or attempt all possible operations with the given numbers without understanding the situation itself (Sowder, 1989).

In contrast to such decorative narratives, a different type of narrative—one that requires the child to adopt a role and act within an imaginary situation—may create conditions for revealing whether counting is used meaningfully. This possibility draws on the well-established understanding of pretend play as the leading activity of older preschoolers, within which key psychological neoformations—imagination and the symbolic function—develop (Vygotsky, 1978; Elkonin, 1978; Venger and Kholmovskaya, 1978; Salmina, 1988). Contemporary research shows that a child becomes engaged in an imaginary play situation when they are required both to act from the character's perspective (to perform the role) and to take into account the specific features of the situation (Veraksa et al., 2023; Veresov et al., 2025; Moraes et al., 2025). This suggests that distinguishing between formal and functional counting mastery may be achieved by creating a situation in which the child can also put him- or herself in the character's place, engaging imagination and symbolization. This hypothesis is grounded in our previous research, which showed that meaningful narratives that invite children to act symbolically on behalf of a character significantly improve the effectiveness of forming early mathematical concepts, particularly in children with low levels of regulation (Veraksa et al., 2022, 2025). However, those studies were not specifically designed to analyze the role of the narrative in distinguishing between formal and functional mastery of a procedure. The present study addresses this gap.

Drawing on the role of pretend play as the leading activity for evaluating the functionality of early mathematical concepts in older preschoolers, we identified five principles for constructing a narrative in assessment tasks:

1. **The task narrative should be built around situations familiar to the child, grounded in his or her everyday or cultural experience**. This is necessary for the child to be able to imagine him- or herself in the situation and adopt the perspective of the character (Veresov and Veraksa, 2024; Ryabkova et al., 2025). Formally, the task is presented to the child through an invitation: “to help,” “to teach someone,” “to show how to do it correctly.” The task thus becomes personally meaningful rather than externally imposed.
2. **The presence of a character with a specific function**. The character should have a stable, understandable mode of action and a desire to fulfill a specific function. From the CHAT perspective, imagination at this age is understood as the ability to put oneself in another's place and see the situation through their eyes (Vygotsky, 2004; Elkonin, 1978). When a child is unable to adopt the character's position, he or she acts exclusively from his or her own frame of reference; such a child does not understand why counting is needed but merely performs a familiar procedure at the adult's request.
3. **The goal as a narratively meaningful outcome**. The goal of the task should not be abstract (“count how many objects are here”) but should be embedded in the narrative: why the character needs to perform the action. For example: “Masha wants to receive guests but does not know whether there will be enough chairs for everyone.” The child, adopting the character's position, internalizes this goal as his or her own.
4. **The mechanism for achieving the outcome is the performance of a mathematical operation**. Importantly, the mathematical operation itself is not explicitly named in the task condition—the child must independently recognize that the character's problem cannot be solved without counting. The character's actions create a substantive gap in the situation: there is a desired state (all guests are fed, gifts are equally shared) and a current state that cannot achieve it without a solution. This gap constitutes the “mechanism”—that which triggers the mathematical operation as a special tool.
5. **The task design should allow differentiation among the levels of counting mastery**. Drawing on the concept of cultural mastery of action (Nezhnov et al., 2011) and Galperin's notion of reasonableness (Galperin, 1966), tasks should be constructed so that performance reveals whether the child is operating at the formal level (mere procedural execution), the reasonable level (orientation to essential conditions), or the functional level (counting used as a tool embedded in another activity). This requires varying the relationship between the narrative and the mathematical operation: from explicit instruction (formal) to explicit operation with object-related traps (reasonable) to implicit operation with conceptual-perceptual traps (functional). We refer to this highest level of mastery as the functionality of counting: the use of counting as a meaningful tool embedded in another activity.

The present study addresses the following *research question*: ≪Can specially constructed story-based tasks, built on these principles, differentiate between formal, reasonable, and functional levels of counting mastery in older preschoolers? And if so, what types of tasks are necessary for assessing each level?≫.

To answer this question, we developed a task battery consisting of three blocks, each designed to assess one of the three levels: formal, reasonable, and functional. The battery was piloted on a sample of older preschoolers.

Beyond examining differentiation among the three levels, we sought preliminary evidence for construct validity. Early mathematical performance is known to correlate with executive functions (working memory, inhibition, cognitive flexibility; Purpura et al., 2017; Zhang et al., 2025), but these studies typically use tasks assessing formal procedural knowledge. If our battery captures different levels of mastery, we would expect different patterns of correlations with executive functions for each block: formal and reasonable blocks should replicate known correlations, as they involve following instructions and maintaining rules, whereas the functional block—if it indeed relies on imagination and decentration rather than cognitive control—should show weaker or no such correlations. This pattern thus provides a way to test the construct validity of the battery.

## 2 Methods

### 2.1 Tasks for assessing the functionality of counting

To assess the functionality of counting—that is, the extent to which counting is used as a meaningful tool rather than a mere procedure—we developed three blocks of tasks. All tasks are built around situations familiar to children (e.g., decorating, collecting items, visiting a park), include a character with a meaningful function (most often a peer of the same age), and present a narratively embedded goal (e.g., helping someone count, dialing a phone number, explaining why different answers were obtained). The blocks differ in the mechanism for achieving the outcome and in how they allow differentiation among levels. Importantly, the formal level serves as the baseline: it indicates that the child has mastered the counting procedure, but it does not yet reveal whether counting is used meaningfully. Only the reasonable and functional levels provide information about functionality—the former showing that the child can apply counting meaningfully when the operation is explicitly called for, and the latter showing that counting has become a tool embedded in another activity (tasks are presented in Table 1):

**Table 1:** Task blocks for assessing the functionality of counting.

Task	Task description	Correct solution criterion
Block 1. Formal		
1.1. Counting forward	Petya wanted to count to 20. He started counting: 1, 2, 3… but lost track. How should Petya continue counting? 1, 2, 3, go on!	Continues counting correctly from where Petya stopped, without returning to 1 (4, 5, 6... 20)
1.2. Counting backward	One boy was learning to count backwards. He started counting like this: 13, 12, 11, 10 … and then he got mixed up. How should he have continued counting? 13, 12, 11, 10, go on!	Correctly continues: 9, 8, 7... without going back to 13
1.3. Numeral recognition	I need to dial this phone number. Please dictate it to me! (The experimenter shows the child a printed slip of paper with numbers 2856013974)	Names all the numbers in sequence without omissions or confusion
Block 2. Reasonable		
2.1. Missing number task	A boy was hanging flags in numerical order (here they are—the experimenter shows a picture with flags from 1 to 9; 2, 4, 5 are missing; 6 and 7 are swapped). Which flags still need to be hung? Where should each one go?	When naming the resulting sequence, notices and corrects the transposition (6 and 7)
2.2. Unitizing of sets as units	Masha collects beautiful buttons. Her mom gave her a box with four buttons! How many buttons does she have now? Here they all are! (the child is shown a picture with two buttons in a row, then a box with the number 4, then another button) Count all of Masha's buttons! Point with your finger as you count	Counts, taking into account the number of buttons in the box, and arrives at 7 *(the experimenter first confirms understanding by asking: “how many buttons are in the box?)”*
2.3. Counting objects (changing counting unit)	The child is shown a picture of 9 blocks (3 cones, 4 cylinders, 2 spheres) in a pile: ≪The children were given a box of blocks. Each of them started counting them—each in their own way. Masha counted: “Four!”—“Correct!” said the teacher. Petya counted: “Three!”—“Also correct!” said the teacher. Vasya counted: “Nine!”—“That is also correct!” said the teacher. What did each child count?≫	Correctly states what each child counted: Masha—cylinders, Petyacones, Vasya—all shapes
2.4. Counting in sets (set comparison)	Three children came to the park, and there a clown was handing out balloons! And not one at a time, but TWO balloons for each child! The clown was holding this many balloons (the experimenter shows a picture with 5 balloons), counted the children, and said: “Oh! There aren't enough balloons! I'll go get more so there will be enough!” How many more did he bring?	Correctly determines how many balloons are missing (e.g., there are 3 children → 6 are needed; 5 are available → 1 is missing)
Block 3. Functional		
3.1. Comparison of discrete quantities	Two rows of five identical buttons each (gray and black) are presented. The child is asked whether there are the same number of buttons in each row. Regardless of the answer, the experimenter explains that Petya adjusted the black row (demonstrating that he spread the buttons apart), and then Nastya said, “What have you done? Now there are fewer gray buttons. You'll have to add some.” She then added two more gray buttons so that the ends of the rows again aligned. The child is then asked whether the rows now have the same number of buttons	States that after the buttons were spread apart, the quantity did not change. After the addition of two buttons, states that there are now more.
3.2. Length comparison	Two straight “paths” made of matches of equal length are presented. The child is asked whether the paths are the same length. Regardless of the answer, the experimenter explains that Petya rearranged the matches in the top row like this (making the path zigzag) and asks whether one path has become longer or whether they are still the same length. Regardless of the answer, the experimenter says, “But Olya said that one path is now shorter, so Petya added one match to it” (a match is added to the top so that the ends of the rows align). The child is then asked whether the paths are now the same length.	States that after the shape of the top path was changed, its length remained the same. After a matchstick was added, states that it became longer.
3.3. Area comparison	Two rugs made of identical small squares are presented (for example, one in the shape of a rectangle and the other in an L-shape or another figure). The child is asked whether the rugs take up the same amount of space or if one takes up more space. Regardless of the answer, the child is told that the rug in the first room was rearranged into a “path” (an elongated strip) so that it would not get in the way of the furniture. The child is then asked which rug now takes up more space.	States that the area did not change despite the change in shape

**Block 1 (3 tasks)—formal level**. Counting serves only as a procedure. The child is asked directly to count or name numerals. There are no traps; this block establishes whether the child has mastered the basic counting procedure.

**Block 2 (4 tasks)—reasonable level**. These tasks assess whether the child orients to the essential conditions of counting. The tasks contain traps that provoke formal solutions (the child must count hidden elements, count in different units, count composite units, or restore missing elements in a numerical series). To succeed, the child must determine what exactly is being counted and in what units, rather than applying the procedure mechanically.

**Block 3 (3 tasks) — functional level**. These tasks assess both orientation to essential conditions and the child's understanding of why counting is needed—what kinds of problems it helps solve more accurately. The child is not instructed to count; counting must emerge spontaneously as a tool for solving the problem. Modified Piagetian conservation tasks were used (discrete quantity, length, and area), in which the child must judge whether the quantity has changed after a transformation.

All tasks were presented within a narrative context, so that the child would not treat them as formal exercises. However, the narrative served a different function in each block: in Block 1 it was merely decorative; in Block 2 it explicitly called for counting while introducing traps; in Block 3 it created a situation where counting was not requested but could emerge as a tool for solving the task.

Quantitative scoring was performed separately for each task based on protocols and audio recordings of children's responses. Each task was scored dichotomously (1—for correct, 0—for incorrect), as the tasks were designed to assess discrete levels of mastery rather than graded performance. Partially correct or ambiguous responses were discussed between the two raters until consensus was reached. All responses were scored independently by two trained researchers with professional backgrounds in psychology (one holding a PhD) and at least 5 years of experience working with preschoolers; inter-rater agreement was high (Cohen's κ = 0.89). Based on task performance, the overall level of counting development (ranging from 0 to 10) was assessed, as well as each of the three levels: formal (0 to 3), reasonable (0 to 4), and functional (0 to 3).

### 2.2 Assessment of executive functions

Executive functions were assessed using the following instruments: subscales from the NEPSY-II (Korkman et al., 2007) were administered to measure inhibition and working memory (both auditory–verbal and visual), and the Dimensional Change Card Sort (DCCS; Zelazo, 2006) was used to measure cognitive flexibility.

## 3 Data analysis

Preliminary analysis using the Shapiro–Wilk test revealed that the distribution of most variables deviated from normality (*p* < 0.05), which necessitated the use of nonparametric methods.

To compare performance across the three task blocks, raw scores were converted to percentage scores (proportion-correct) to account for the different numbers of tasks in each block. Pairwise comparisons between blocks were conducted using the Wilcoxon signed-rank test with Bonferroni correction (critical alpha set at *p* < 0.017). To examine transitions between levels, children were classified as successful or unsuccessful on each level based on predefined criteria. Crosstabulations with Fisher's Exact Test were used to examine the hierarchical relationship between levels.

To assess construct validity, Spearman's rank correlations were calculated between percentage scores on each block and executive function measures (working memory, inhibition, cognitive flexibility). Statistical analyses were performed using IBM SPSS Statistics, version 26.

## 4 Sample

The sample consisted of 56 older preschoolers (41.1% of boys, Mage = 68.9 months, SDage = 3.86). Children were recruited from two public kindergartens in Moscow. Inclusion criteria were: (a) age between 5 and 6 years, (b) typical development (no diagnosed developmental disorders), and (c) parental consent for participation. No exclusion criteria beyond these were applied. The sample reflects a convenience sample from the participating kindergartens, which served families from middle to upper-middle socioeconomic backgrounds.

The assessment was conducted in two sessions. The mathematics battery took approximately 20 min per child, and the executive function assessment took approximately 30 min. The order of task administration was fixed across all children: first the mathematics tasks, then the executive function measures. All assessments were administered individually in a quiet room at the kindergarten during the morning hours by trained psychologists with experience in preschool assessment.

No sex differences were found on any of the assessed measures (Kruskal–Wallis test).

## 5 Results

### 5.1 Descriptive statistics

Descriptive statistics for the three task blocks are presented in Table 2. The highest mean percentage score was observed for the formal level (*M* = 67.26%, SD = 33.9), followed by the reasonable level (*M* = 34.82%, SD = 22.7), and the lowest for the functional level (*M* = 19.64%, SD = 23.6).

**Table 2:** Descriptive statistics for the three task blocks (percentage scores).

Level	*N*	Mean (%)	SD	Min	Max
Formal	56	67.26	33.9	0	100
Reasonable	56	34.82	22.7	0	100
Functional	56	19.64	23.6	0	100

The assessment of executive functions showed that the mean values for all components were within the age norms established for Russian preschoolers aged 5–6 years (Almazova et al., 2024), suggesting that the sample did not show marked deviations from available age norms on these measures.

### 5.2 Hierarchical relationship between levels

To examine the hierarchical relationship between the three levels of counting mastery, children were classified as successful or unsuccessful on each level based on the following criteria: formal—≥67% correct (2 out of 3 tasks); Reasonable—≥75% correct (3 out of 4 tasks); Functional—≥67% correct (2 out of 3 tasks). Crosstabulations with chi-square tests were used to examine transitions between levels. The proportion of children who succeeded decreased systematically from the formal level (42.9%, *n* = 24) through the reasonable level (10.7%, *n* = 6) to the functional level (1.8%, *n* = 1).

Crosstabulation analysis revealed a strict hierarchical pattern. All children who succeeded on the reasonable level (*n* = 6) also succeeded on the formal level. Similarly, the only child who succeeded on the functional level also succeeded on both the formal and reasonable levels. No children succeeded on a higher level without also succeeding on the preceding level(s). Fisher's Exact Test confirmed a significant association between formal and reasonable success (*p* = 0.004). Due to the small number of children who succeeded on the functional level (*n* = 1), the associations involving functional success did not reach statistical significance (Formal–Functional: *p* = 0.429; Reasonable–Functional: *p* = 0.107).

These findings confirm that the three levels form a strict hierarchy: formal mastery serves as a prerequisite for reasonable counting, which in turn serves as a prerequisite for functional use of counting as a tool embedded in another activity. However, only 6 out of 24 children (25%) who mastered the formal level also demonstrated reasonable counting, and only 1 out of 6 (16.7%) who mastered the reasonable level also demonstrated functional counting. This suggests that each level represents a qualitatively distinct step in the development of counting mastery, and that success at a lower level does not guarantee success at the next level.

### 5.3 Assessment of the construct validity of the battery

To examine the construct validity of the battery, we calculated Spearman's rank correlations between performance on each task block (percentage scores) and executive function measures (working memory, inhibition, cognitive flexibility). The results are presented in Table 3.

**Table 3:** Significant Spearman correlations between levels of counting functionality and executive functions.

Level	Executive function measure	*r*_*s*_	*P*
Formal	Visual working memory	0.284^*^	0.034
	Auditory-verbal working memory	0.301^*^	0.027
	Inhibition (naming)	0.340^*^	0.022
Reasonable	Visual working memory	0.407^**^	0.002
	Auditory-verbal working memory	0.372^**^	0.006
	Inhibition (naming)	0.356^*^	0.016
	Inhibition (inhibition)	0.353^*^	0.019
Functional	*None*	—	—

^*^p < 0.05; ^**^p<*0.01*.

As expected, performance on the formal block correlated significantly with visual working memory (*r*_*s*_ = 0.284, *p* < 0.05), auditory-verbal working memory (*r*_*s*_ = 0.301, *p* < 0.05), and inhibition (naming: *r*_*s*_ = 0.340, *p* < 0.05). Performance on the reasonable block showed significant correlations with visual working memory (*r*_*s*_ = 0.407, *p* < 0.01), auditory-verbal working memory (*r*_*s*_ = 0.372, *p* < 0.01), inhibition (naming: *r*_*s*_ = 0.356, *p* < 0.05), and inhibition (inhibition: *r*_*s*_ = 0.353, *p* < 0.05). No significant correlations were found between the functional block and any of the executive function measures (all *r*_*s*_ < 0.30, *p* > 0.05).

This pattern supports the construct validity of the battery: performance on the formal and reasonable blocks replicates the well-established association between early mathematics and executive functions, while the absence of correlations with the functional block suggests that functional counting taps into different mechanisms, consistent with our theoretical framework. However, this interpretation is preliminary and requires further investigation with direct measures of imagination and decentration.

Thus, the developed task battery differentiates preschoolers by their level of counting functionality: performance decreased systematically from the formal to the reasonable to the functional level. The pattern of correlations with executive functions—significant for the formal and reasonable levels, but absent for the functional level—is consistent with the theoretical framework and provides preliminary evidence for the construct validity of the battery. However, further research with larger samples and direct measures of imagination and decentration is needed to confirm this interpretation.

## 6 Discussion

The aim of this pilot study was to develop and test a task battery for assessing the functionality of counting in older preschoolers—that is, the extent to which counting is used as a meaningful tool rather than a mere procedure. Drawing on CHAT (Vygotsky, 2004; Elkonin, 1978; Nezhnov et al., 2011; Galperin, 1966; Veraksa et al., 2023; Veresov et al., 2025), we distinguished three levels of counting mastery—formal, reasonable, and functional—and identified five principles for constructing narrative-based tasks: grounding in the child's experience, a character with a meaningful function, a narratively embedded goal, an implicit mathematical operation, and differentiation among the three levels. The battery was designed to assess these levels through three corresponding task blocks, each built according to these principles.

The results provide preliminary evidence that the battery successfully differentiates among the three levels. The proportion of children who succeeded decreased systematically from the formal level (42.9%) through the reasonable level (10.7%) to the functional level (1.8%). Moreover, the hierarchical analysis confirmed a strict pattern: all children who succeeded on the reasonable level also succeeded on the formal level, and the only child who succeeded on the functional level also succeeded on both preceding levels. This pattern is consistent with the theoretical framework, which posits that formal procedural mastery serves as a baseline for reasonable counting, which in turn serves as a prerequisite for functional use of counting as a tool embedded in another activity (Nezhnov et al., 2011).

The pattern of correlations with executive functions allows for an important conclusion. While performance on the formal and reasonable levels was predictably associated with working memory and inhibition—replicating the well-established link between early mathematics and executive functions (Purpura et al., 2017; Zhang et al., 2025)—the functional level showed no such correlations. This means that success in tasks where counting serves as a tool embedded in another activity is not determined by the level of cognitive control. Consequently, relying solely on the development of executive functions is insufficient for fostering functional mathematical understanding. The critical condition is creating situations in which children learn to use counting as a meaningful tool for solving real-world problems, rather than as a procedure to be performed upon instruction. This finding aligns with the CHAT perspective, according to which the quality of mastery—whether an action remains a separate operation or becomes a tool serving another activity—matters no less than the level of cognitive development.

The findings have implications for educational practice. The battery provides a tool for teachers and educational psychologists to assess not only whether a child can count, but how counting is used—whether as a mere procedure, as a reasonable means in familiar situations, or as a functional tool embedded in other activities. This differentiation is important because it moves beyond simple procedural assessments and helps identify children who may need support in understanding the purpose of counting in real-life contexts (Purpura et al., 2017; Zhang et al., 2025; Chatzaki et al., 2024). Moreover, the principles of task design—grounding tasks in familiar situations, including a character with a meaningful function, embedding a narratively meaningful goal, and making the mathematical operation implicit—can inform the development of diagnostic and educational materials for early mathematics education (Chatzaki et al., 2024; Vessonen et al., 2023). This aligns with contemporary research showing that embedding learning in meaningful play contexts supports the development of cognitive functions in preschoolers (Shiyan, 2025; Shiyan and Korotun, 2024; Veresov et al., 2025).

## 7 Limitation and future directions

Several limitations should be acknowledged. First, the sample size was relatively small (*N* = 56) and local, which limits the generalizability of the findings. Second, the tasks within each block were designed to capture different facets of the same level of counting mastery rather than to form a homogeneous scale. This means that the total block scores should be interpreted as indicators of performance across a range of situations within that level, rather than as measures of a single underlying construct. This design choice limits the use of internal consistency measures such as Cronbach's alpha but was intentional: it allowed us to cover a broader range of situations in which counting might be used formally, reasonably, or functionally. Third, we used dichotomous scoring, which may have reduced sensitivity to partial mastery. Fourth, we did not directly measure imagination or decentration, so the interpretation of the absence of correlations between the functional block and executive functions remains tentative. Fifth, although inter-rater reliability was high, the study was not designed to assess test-retest reliability or other psychometric properties.

Promising directions for future research include: (1) validating the battery on larger and more diverse samples, (2) incorporating direct measures of imagination and decentration to test the theoretical interpretation of the functional level, (3) developing a formative experiment in which the proposed principles of narrative construction are used not only for assessment but also for teaching functional counting, and (4) adapting the instrument for assessing other mathematical operations and for other age groups.

## 8 Conclusion

1. The developed task battery successfully differentiates among three levels of counting mastery in older preschoolers: formal, reasonable, and functional. The proportion of children who succeeded decreased systematically from the formal level (42.9%) through the reasonable level (10.7%) to the functional level (1.8%).
2. The hierarchical analysis confirmed a strict pattern: all children who succeeded on the reasonable level also succeeded on the formal level, and the only child who succeeded on the functional level also succeeded on both preceding levels. This confirms that formal procedural mastery serves as a baseline for reasonable counting, which in turn serves as a prerequisite for functional use of counting as a tool embedded in another activity
3. The pattern of correlations with executive functions provides preliminary evidence for the construct validity of the battery. Performance on the formal and reasonable levels correlated with working memory and inhibition, replicating known findings in the literature. The absence of such correlations for the functional level suggests that functional counting may rely on different mechanisms—likely imagination and decentration—although direct measures of these processes are needed to confirm this interpretation.
4. The battery provides a tool for teachers and educational psychologists to assess not only whether a child can count, but how counting is used—whether as a mere procedure, as a reasonable means in familiar situations, or as a functional tool embedded in other activities. This differentiation helps identify children who may need support in understanding the purpose of counting in real-life contexts.
5. The five principles of narrative construction—grounding in the child's experience, a character with a meaningful function, a narratively embedded goal, an implicit mathematical operation, and differentiation among levels—can inform the development of diagnostic and educational materials for early mathematics education.

## Data Availability

The original contributions presented in the study are included in the article/supplementary material, further inquiries can be directed to the corresponding author.
